# Metal Marking Behavior and Testing of Porcelain Tableware

**DOI:** 10.3390/ma15072442

**Published:** 2022-03-25

**Authors:** Luc Hennetier, Ana Moura, Manuel Ribeiro

**Affiliations:** 1 Technological Center for Ceramic and Glass (CTCV), 3040-540 Coimbra, Portugal; luc@ctcv.pt (L.H.); ana.moura@ctcv.pt (A.M.); 2 Materials Research and Development Center (UIDM), Polytechnic Institute of Viana do Castelo (IPVC), Rua Escola Industrial e Comercial de Nun’Álvares, 4900-347 Viana do Castelo, Portugal

**Keywords:** metal marking, scratch resistance, porcelain tableware, glaze, cutlery, crystalline particles, zircon, testing

## Abstract

The term “metal marking” is widely used to define the common phenomenon of tableware glazes being damaged by metallic cutlery. Appearing as unaesthetic gray marks and scratches resulting from normal conditions of use, these defects deeply affect the performance of ceramic products, especially in intensive environments, such as in the hospitality industry. The scope of this article is to establish a comprehensive review of the phenomenon, focusing on the physical and chemical mechanisms involved in the process, and their interactions and consequences. It also intends to list the different methods normally followed to avoid or at least reduce this defect, in order to enhance the durability of porcelain dishware. This manuscript also provides a review of the different testing methods developed and used by the tableware industry and technical centers to quantify the ability of porcelain tableware to produce metal marks. To face the current lack of any international or at least national standard testing procedure that would permit a reliable comparison of products, a new metal marking test developed at the Technological Center for Ceramic and Glass (CTCV) is presented as an alternative to common tests normally based on knives as a marking tool.

## 1. Introduction

**Figure 1:**
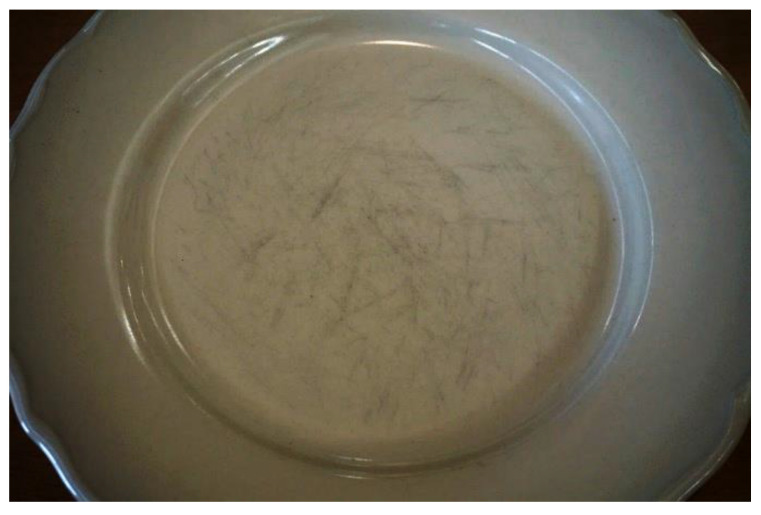
Metal marks on a dinner plate.

It is common to observe, in the bottom of ceramic plates or cups, where food and liquids are placed, unattractive gray or dark marks that are characteristic of regular contact with metal knives, spoons or forks with the product surface (Figure 1). 

These marks can also be accompanied by small cracks of the glaze and even scratches [1,2]. Scratches normally occur at a later stage of use and are responsible for the gradual increase of glaze deterioration. The roughness increases as some glaze fragments are pulled from the surface; this is particularly inconvenient as it may not allow a correct cleaning/hygienization of the dish due the possibility of harboring dirt and bacteria. This creates an aesthetic degradation of the ceramic tableware, which depends on intensity of use, and may significantly reduce the useful lifetime of products. The reasons for complaints from consumers are more related to the unsightly metal marking effect than to cracks and scratches, which tend to appear at a much later stage of use than metal marks. This problem brings higher costs especially in the hospitality industry—e.g., hotel, restaurants, and coffee houses—which are normally driven by high quality standards and where the ceramic tableware is exposed to intense cycles of use (serving and washing).

Producers are occasionally confronted with this type of situation, and the mechanisms underlying this phenomenon have been the subject of a few in-depth scientific studies [1,2,3,4,5,6]. The different works available on the subject focus on the study of the mechanisms and on the development of quantitative assessment methodologies, through simulation tests of marking and washing. They all highlight the persistence of a poorly understood and cross-cutting problem in the tableware industry [1], as it affects all kind of ceramic tableware glazes, independently of the ceramic paste, whether porcelain, bone china, stoneware, or even earthenware. It should be noticed that although it is a very common problem, deeply related to material science and mechanics, the reason few studies are available is related to the fact that most of research and development works are maintained confidential, since tableware companies in partnership with glaze companies carry them out. Apart of being private, developed solutions or improvements are frequently restricted to a specific product and glaze composition and hence difficult to transpose to other glazes and other companies.

Thus, this work focuses first on the physical mechanisms, which are considered to be responsible for the glaze deterioration, from metal particles deposition onto the glazes surface to the development of cracks and scratches. It intends to explain how the physical and chemical characteristics of glazes and metallic utensils affect the performance regarding metal marking, but also to highlight the interrelationship between the several phenomena occurring during the progressive degradation of the tableware surface, especially glaze aging from mechanical and chemical stress.

Ceramic tableware is an expanding market that includes all ceramic dishes, cups, and bowls mainly used for catering services and for domestic purposes. A representative part of the ceramic tableware production is in fact absorbed by the food service industry sector—HOtels, REstaurants and CAtering—generally abbreviated with the HORECA acronym, widely used in European countries. This sector is extremely important for ceramic producers and is characterized by high and rigorous quality requirements, forcing companies to adapt and/or develop solutions in order to fulfill the final customer’s needs in terms of design, aesthetics, performance, and functionality. In fact, ceramic products used in this sector are subjected to intensive use and subsequently exposed to frequent attack, such as metal marking, mechanical wear-especially on the rim, due to repeated shocks during manipulation, but also on the plate bottom due to handling and stacking-and surface degradation caused by the aggressive washing with chemical detergents. Consequently, in order to enter or to strengthen their position into these strategic markets, companies are continually working on the development of new glazes to provide new designs and colors (such as reactive glazes) and, at the same time, to obtain better performance, especially concerning the metal marking occurrence. The growing importance of metal marking is shown by major retailer’s efforts to develop in-house tight test procedures and acceptance criteria of products about their performance toward contact with cutlery. This necessity is actually due to the glaring absence of an international metal marking standard test. In its recent specification for domestic and hospitality use of ceramic tableware (BS8654:2015—Domestic and hospitality use ceramic tableware articles intended for contact with foodstuffs—Specification ) [7], the British Standards Institution was the first standards developing organization to include metal marking resistance as a performance criterion, but the evaluation is still performed following a non-standardized in-house method developed by a national laboratory (Lucideon, Stoke-on-Trent, UK).

This lack of standardization led to a wide variety of internal procedures, developed by companies, technical laboratories, or even customers. Together with the subjectivity of samples evaluation, it prohibits:

- An adequate comparison between products tested according different methods
- The development of an accurate and reliable database to provide technical information on the performance of products.

As a standard test method to determine the metal marking resistance is needed to evaluate the performance and the behavior of ceramic tableware when in contact with cutlery, a review of usual tests attributes showing their limitations is then presented. As a result of the knowledge acquired in Research and Development projects for development of improved metal marking resistance tableware and respective characterization, a new test was implemented at Technological Center for Ceramic and Glass (CTCV) in order to reduce the drawbacks and subjectivity of actual available tests and which is already used by some national companies. The standardization process at Portuguese national level is in progress.

## 2. Metal Marking Mechanism

A first mention of the phenomenon known as metal marking can be found in scientific literature in the early 20th century [8]. In this article, the authors described that metallic objects (e.g., silver-plated or steel-based knives) that repeatedly slide across the surface of any kind of ceramic tableware are able to leave some marks that are not always removable. These gray marks are similar to a line made with a pencil on a white paper sheet, as a result of metal particles deposition due to the regular contact of cutlery with ceramic surface.

Presently, the term “metal marking” normally refers to the gray or dark lines, but also encompasses the damages caused to the glaze, including cracks and scratches resulting from the use of metal utensils (knives but also forks and spoons) [2]. Thus, the metal marking phenomenon can be described according to the two effects resulting from the contact between cutlery and tableware:

- **Metal marks**: Deposition of steel (or aluminum, silver) particles from a metallic kitchen or table utensil (knife, fork, or spoon) susceptible to lose matter through mechanical action (sliding/frictional wear) on the tableware surface susceptible to fix these particles. Generally, smooth metal particles can be durably fixed onto the surface and thus difficult to remove, while harder particles have more difficulty to adhere to the surface and can be removed more easily. This problem tends to happen when the glaze is rougher and harder (but not mandatory) than the cutlery material and the gravity of the defect depends on the force applied with the utensil.
- **Scratches and cracks**: Degradation of the glaze surface under the mechanical action of cutlery made of harder material (friction) and/or under high applied pressure. Cracks and loss of glaze material (chipping) create some roughness and/or decoration degradation of the tableware, and can also cause the pulling out and deposition of metallic particles on the glaze asperities. Scratches and cracks cause irreversible surface deterioration.

**Figure 2:**
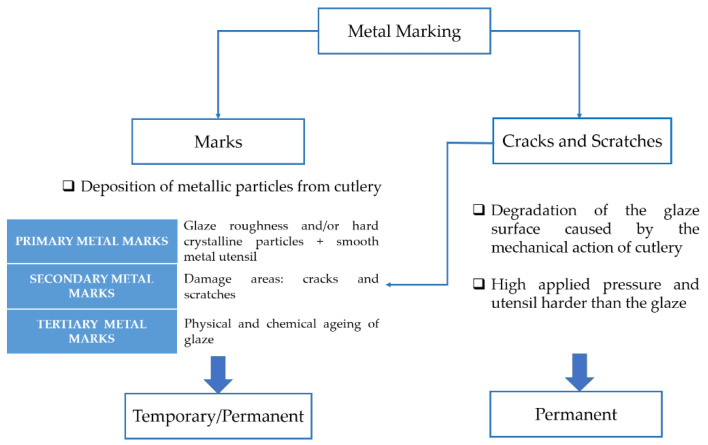
Metal marking definition.

**Figure 3:**
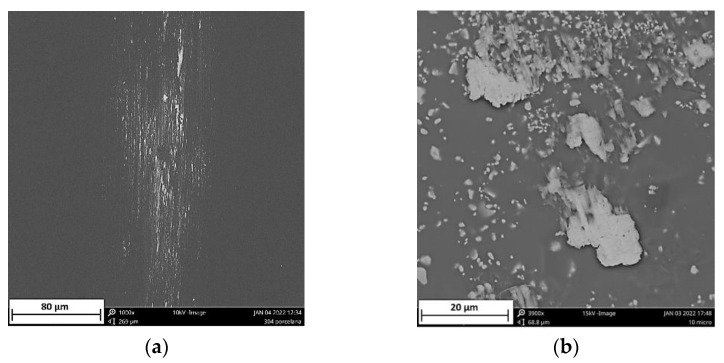
SEM micrographs of primary metal marking (**a**) metal marks from smooth utensil sliding over on porcelain hard glaze (**b**) metal tearing from protruding zircon particles present in opaque glaze.

**Figure 4:**
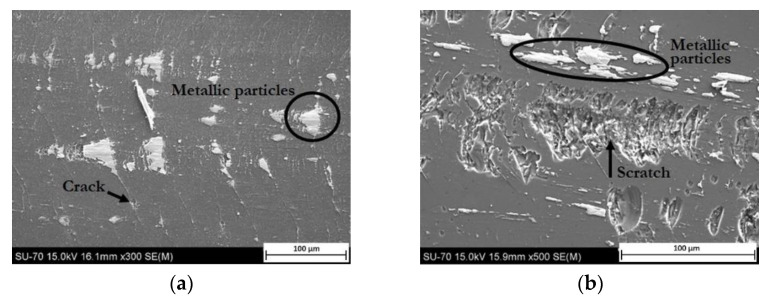
SEM images from glaze surfaces (**a**) earthenware plate after metal marking test (deposition of metallic particles in the cracks); (**b**) extensively used porcelain plate (deep scratches and metal marks resulting from normal use are presented).

A schematic description of metal marking phenomenon is presented in Figure 2, and micrographs of metal marked surfaces obtained by scanning electron microscopy (SEM,) illustrate the mechanism at microscale level in Figure 3 and Figure 4 showing the interdependence of both effects. On all the pictures, metal deposits (white areas) can be observed on the surface of the glaze, as well as different levels of structural damage.

The main reasons generally mentioned for metal marks are the roughness and hardness of glazes surface and the presence of hard crystalline particles in the glaze, coming from recrystallization processes during firing or already included in the glaze composition for special purposes, such as zircon in opaque glazes [1,2,9]. Local heating during abrasion also contributes to the bonding of torn out particles to the surface [3]. These kinds of marks, called **primary metal marks**, are illustrated by SEM in Figure 3a where metal particles from a smooth utensil are deposited on a hard porcelain glaze, and in Figure 3b where metal is torn out when passing over zircon particles embedded at the surface of an opaque glaze.

Even on a relatively new glaze, the pressure applied during the movement of the utensil can be strong enough to start damaging the glaze, forming Hertzian cone cracks. As it can be seen (Figure 4a), these cracks create sharp edges that promote the removal of metallic particles from cutlery [10] and metal build-up at cracks edge. When the glaze is becoming more damaged (Figure 4b), particles are removed from cracks weakened area (chipping), and cracks start to be visible to the naked eye. The dramatic increase of roughness and sharp edges led to higher metal deposition in these rugged areas, where the removal is even more hindered. Marks resulting from the progressive degradation of the glaze by cutlery will then be referred as **secondary metal marks**. Scratches with other origins also contribute to this kind of mechanism. In particular, scratching resulting from the stacking of the plates, as the rough unglazed foot of the plate wears out the plate bottom where utensils will slide on afterwards [3].

Mechanical properties of the glaze are definitely essential to understand the cracking and scratching mechanisms. However, studies showed that there are a wide range of parameters that have to be taken into account, considering that several attributes of both marked object and marking utensil influence directly the phenomenon, listed in Table 1 and Table 2. Apart from already mentioned glaze hardness and roughness, thermal expansion coefficient (TEC) of both glaze and body are important to look at in order to understand the crack occurrence and propagation [11]. The local rupture and subsequent propagation of crack occurs when applied pressure overcome the compressive stress of the glaze, which depends on the TEC mismatch between glaze and body [12]. The presence of microdefects in the glaze, even in the near surface, should be taken into account, as shown by Heo et al. [13] who studied the negative influence of pore structure and distribution in the glaze on wear resistance of glazes.

In the early 1930s, Geller and Creamer [8] published a first study where they investigated the influence of sulfur ions in the furnace atmosphere on glaze roughness degradation and subsequent poor metal marking performance, showing this problem has been worldwide for a long time. Further studies were focused mainly on metal marking description [1,2], and on the attempt of making a deeper relation between this phenomenon and the glaze properties—composition, thermal treatment, mechanical properties, superficial defects, and texture.

The nature of glaze influences directly the metal marking behavior. Matte and opaque glazes, consisting of well-dispersed crystalline phases in the vitreous matrix, are easily marked due to their inherent roughness but, on the other hand, they are normally relatively easy to clean. The resistance to primary metal marking of glossy glazes depends on the level of glaze opacity, directly related to the quantity of opacifiers crystals in the glaze [1,2,3]. On the contrary transparent glazes, characterized by crystalline phase inexistence, are more resistant to primary metal deposition.

Type and size of crystal phases used as opacifiers—such as ZrO_2_, SnO_2_ and TiO_2_—have an influence on metal marking resistance [5]. For instance, zircon (ZrSiO_4_) is an excellent, economical opacifier agent widely used in ceramic industry, due to its high refractive index. However, its presence in opaque glazes is largely associated with primary metal marking resistance [1]. The introduction of ZrSiO_4_ into glazes can be made by addition of zircon particles into the raw glaze, or through the formation zircon during firing from zirconium-based compounds such as zirconia (ZrO_2_). It influences directly the presence and morphology of zircon at the glaze surface, as several studies of zircon crystallization during firing shows [9,14,15]. As at lower levels, zircon is mostly dissolved in glass matrix, higher amounts are needed to achieve the necessary level of opacity [2]. In this case, zircon tends to partially dissolve and then to recrystallize, with formation of rounded shaped zircon crystals clusters anchored at the surface. With zirconium-based frit, well-dispersed acicular crystals are obtained, generally aligned with the surface. In both cases, metal marking behavior is negatively affected by the presence of those hard particles at the surface (Figure 3b).

As for zircon, the presence of devitrification areas or small crystals on the glazed surface affects the friction coefficient and is responsible for a higher hardness than the one observed in cutlery. Indeed, the presence of crystalline particles on the glazed surface locally alters the microhardness of the glaze structure, as the vitreous matrix hardness varies normally between 350 and 750 Hv (Vickers hardness) [5,12]. Zircon (>800 Hv) [16], quartz (1100–1200 Hv) [17] or cristobalite (±1000 Hv) act as abrasive agents for cutlery, whose hardness ranges normally between 300 and 700 Hv [4,10]. Quartz is a normal component including glaze raw materials, the optimization of the particle size may play a positive role in reducing metal marking [5], as long as other properties (such as whiteness) are compensated. As cristobalite forms during glaze devitrification, glaze system and especially firing curve are normally studied and optimized to reduce its occurrence.

**Figure 5:**
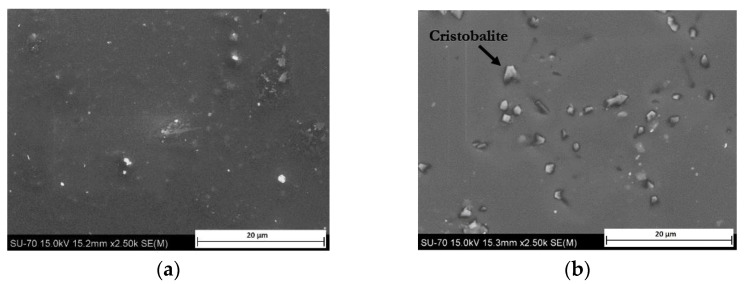
SEM images from the glaze surface of a new porcelain plate (**a**) before; (**b**) after accelerating chemical aging.

Unlike primary and secondary metal marking, **tertiary metal marking** occurs at a much later stage of the tableware lifetime, and arises from the progressive degradation of glaze properties due to prolonged use (contact with food, washing with detergents). In particular, repeated washes with detergents (mostly alkaline), quite aggressive, mainly used in the hospitality industry, promote aging of the glaze, demonstrating that the alkali attack the silicate glass matrix, leaving the crystals unchanged [12]. It has also been shown that even less aggressive detergents (low-alkaline) are corrosive for tableware glaze [18]. The effective wear of the vitreous matrix and the higher density of unaltered crystals cause an increase in surface roughness and hardness modifying the resistance behavior to metal marking. SEM micrographs presented in Figure 5 show an example of porcelain glaze chemical aging.

Figure 5a shows the surface of a new porcelain plate. The surface is relatively smooth, with a 0.1 Ra roughness typical for that product category, and some crystalline particles can be discerned into the glaze matrix, mainly under the surface. The same porcelain plate was exposed to an accelerating aging procedure following EN 12875-4 - Mechanical Dishwashing Resistance of Utensils—Part 4: Rapid Test for Domestic Ceramic Articles [19], which allows testing the dishwashing resistance of ceramic articles for domestic use. After the test, the analysis by SEM (Figure 5b) and X-Ray diffraction revealed an altered surface, showing an erosion of the silicate glass matrix leaving cristobalite particles unaltered and anchored at the surface, thus appearing acting like a polishing cloth, following thus the principle of primary metal marks.

## 3. Improvement of Metal Marking Performance

Efforts of tableware companies and glaze suppliers to obtain better performance to metal marking are essentially based on:

- Optimization of process and glaze compositions, or
- Protection of glaze surface by thin coatings.

As a first approach, developments in producing environment are based on replication of glazes with excellent metal marking performance, even if, in most cases, the reason for its good behavior remains elusive. As companies normally own hundreds of glazes due the wide variety of colors, effects and final aspects, it is worth trying to take advantage of the composition frame of a good glaze for an extended range of formulation with different additive and pigments necessary to provide the final glaze aesthetics. Some adjustment of glaze and even body composition may also be made in order to optimize the hardness of the vitreous phase, working on the silica content for instance [5], or on the glaze tension status. Except for crazing glazes which are characterized by an aesthetic net of cracks, obtained due to the tensile status of the glaze when TEC of the body is higher than the glaze TEC, glazes are normally in controlled compression status. The difference in glaze and body thermal expansion may be raised, but up to a certain limit in order to avoid glaze peeling defect.

As already mentioned, developments based on glaze formulation are conducted in a way to reduce the crystalline particles incorporation or formation: the firing process modification to limit the cristobalite crystallization, the particle size reduction of quartz [5], and the research of economical alternative to zircon. Even if the latter is quite challenging as alternatives are normally expensive (tin oxide) or ineffective (alumina is harder than all others raw materials) from the point of view of the metal marking inhibition. 

The main methodologies followed to improve products are summarized in Table 3.

Despite a lack of information disclosure for commercial purposes from companies, some recent studies showed several interesting developments for glazes improvements. In their study, Pee [6] improved the glaze properties by adding low melting point frits (ZnO and B_2_O_3_) in a traditional celadon glaze and by controlling the reducing fired atmosphere, with the objective to reduce the roughness of the glaze and increase its hardness. The high melting point of celadon glaze leads to low viscous behavior, causing high surface roughness, and thus poor performance in metal marking. They observed a combination of surface roughness decrease and a hardness increase, as the frit content increased and the atmosphere reduced, respectively. Wear resistance tests using a stainless steel ball performed in order to evaluate metal marking resistance showed a strong reduction in metal marking behavior with increasing frit content, resulting from the reduction of friction coefficient. At the same time, the higher hardness led to a lower tendency to crack and scratch.

In another study, Güngör and Altun [5] investigated the influence of quartz particle size on scratch resistance of soft porcelain opaque glazes, fired at 1250 °C, normally characterized by a lower hardness. The reduction on quartz particle size leads to a higher dissolution of quartz in the glass matrix, and consequently a lower quartz content in the glaze after firing. The verified improvement of metal marking performance—realized with a steel knife—with the quartz particle size decrease was related with the smoothness of the glaze surface, as already showed by Pee [6]. Nevertheless, it should also be considered that the increase in glaze hardness, as the article suggests, also contributes to avoid secondary metal marks resulting from glaze degradation under the pressure applied by the knife.

Some solutions based on technology developed not specifically for metal marking, but for scratch resistance, chemical resistance, and easy-cleaning purposes, for instance, are sometimes applied with a metal marking reduction purpose. The main drawback is the durability of such solutions, as they are normally based on an organic matrix loaded with functional nanoparticles. The limited duration of effects and price restrict the spread of these products in the industry, as well as some concerns about health consequences. Inorganic thin layers, based for instance on sol–gel techniques, are also investigated, namely for surface cleanability—associated with surface smoothness—but their application is limited due to the additional costs and also difficult industrial implementation in more traditional industries, such as tableware companies [20].

## 4. Metal Marking Testing Procedure

**Figure 6:**
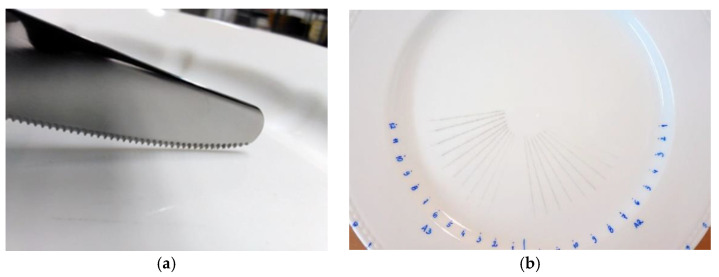
Metal marking definition (**a**) a knife contacting with a glaze (**b**) porcelain plate after metal marking test, on which it is possible to observe the presence of gray marks.

**Figure 7:**
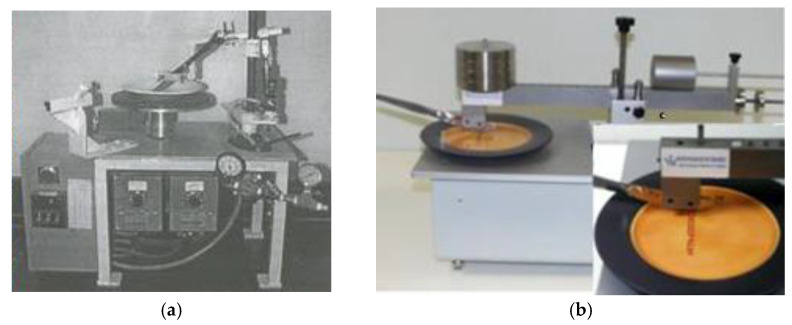
Examples of metal marking apparatus used by some research centers (**a**) NYSCC—New York State College of Ceramics [2] and (**b**) SFC—Société Française de Céramique [4].

A metal marking test aims to reproduce and simulate the interaction between a metallic object that is drawn across the glazed surface, reproducing the food cutting movement performed by a knife, or a spoon stirring the coffee into a cup for example, in a laboratory environment under controlled conditions. Metal marking resistance is a crucial parameter to evaluate the performance of ceramic tableware and as it is one of the most required specifications from retailers and customers, it is inconceivable that no standardized method is currently available. Consequently, ceramics companies are obliged to develop their own routine tests to control their daily production, as well as research and development laboratories for investigation purposes [1,2,3]. Some research works developed qualitative assessment methodologies, through simulation tests of marking and washing methodologies to be used by companies [4,10]. Usually metal marking tests involve a commercial metallic utensil (cutlery) moving back and forth on the surface of the ceramic tableware (Figure 6a) followed by qualitative visual surface inspection (Figure 6b), and optionally completed by a dishwasher resistance followed by a complementary metal marking test in order to assess the impact of chemical aging on performance. Figure 7 shows two typical equipment setups used to perform the metal marking tests.

To date, the BS 8456:2015 specification about domestic and hospitality use of ceramic tableware articles intended for contact with foodstuffs [7] contains the closest official requirement for metal marking performance, despite standardization procedure attempts by the ASTM committee in the 1980s [3] and eventually in other countries. In this specification, a qualitative classification scheme is referred, related to a specific in-house test developed by Lucideon. The test uses a stylus, instead of knives or other utensils, made from different common cutlery grades of stainless steel at different applied loads. The ceramic tableware is labeled according to the level of marks: distinct marks (they cannot be removed by cleaning)—classification 2; slight marks removable by cleaning—classification 1, or absence of marks—classification 0. According to the referred specification, an item is suggested to be metal marking resistant if it obtains classification 0 or 1.

**Figure 8:**
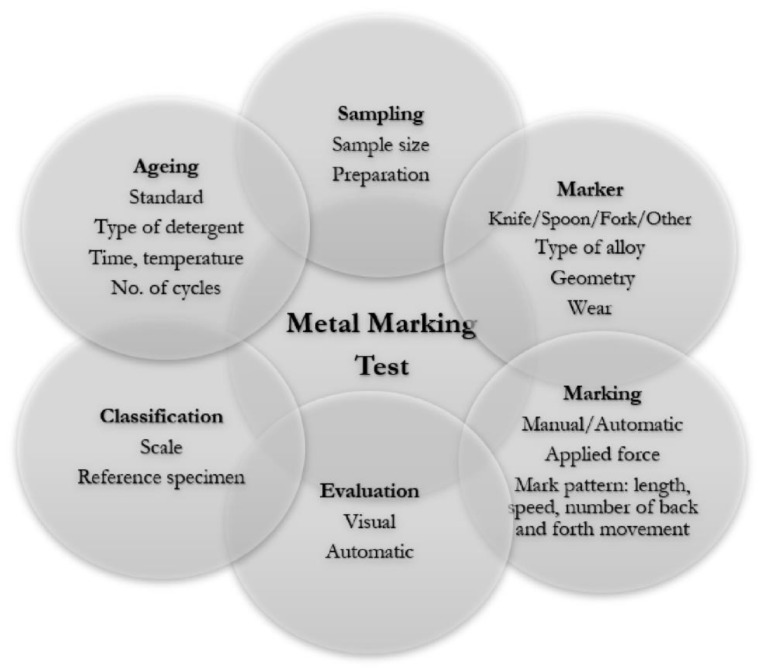
Set of parameters to take into account for a metal marking test.

Our knowledge and practice of a wide range of existing in-house private tests established by companies and retailers-that cannot be disclosed publically show us that it is not worth it to compare products tests following different procedures and evaluation criteria. Thus, a uniform methodology is needed to be clearly set out to perform a metal marking test. A precise definition of a large set of parameters (Figure 8) will guarantee reproducibility and repeatability.

Despite the existence of different test protocols used by different entities, the steps to be followed are similar and are related to:

1. Sample preparation (tableware selection, washing and drying).
2. Glazed surface marking with a metallic utensil: knife, spoon, fork or even a steel ball.
3. Marks and scratches evaluation (qualitatively) and classification.

Optionally,

4. Aging procedure following dishwashing resistance test.
5. Additional surface marking, evaluation, and classification.

### 4.1. Sample Selection

**Figure 9:**
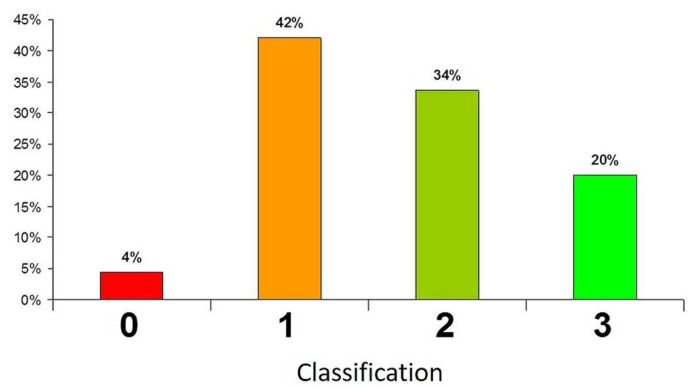
Metal marking classification distribution obtained on large batch of ceramic stoneware products (0—severe marking, 1—distinct marking, 2—slight marking and 3—no marking).

Sample size is not always specified by procedures. In most cases, the test is realized on no more than three specimens, which is clearly not representative of the normal fluctuation of final glaze properties, considering that even if industrial furnaces’ temperature control and homogeneity are becoming better and better, it is normally considered that each item loaded in the furnace has its own thermal history. Thus, samples must be representative of the production batch, in order to obtain representative results. In a project involving CTCV on the development of improved glaze for metal marking, it was decided to test all the plates fired during the same firing cycle, with the same glaze, considering five cars in a row, representing 450 plates. The metal marking test followed included a final evaluation based on a 4-scale classification (0—severe marking, 1—distinct marking, 2—slight marking and 3—no marking). The final results showed a wide dispersion of results, with around half of the plates considered resistant to metal marking (classification 2 and 3) and the other half rejected (Figure 9). The study also gave valuable information on the influence of the position of the articles on the car and thus their proximity to the gas burner, showing preferential placing areas for better metal marking behavior. These results clearly showed that a metal marking test cannot be considered trustful when performed on less than three random specimens.

### 4.2. Sample Preparation

Normally tableware is simply washed with water and detergent and cleaned with a cloth or dried in oven. Nonetheless a careful cleaning of samples before metal marking test is required, in order to eliminate any possible contaminations (dust particles or grease). Karlsson et al. [21] showed the influence of surface cleaning on wear behavior, especially contaminations such as grease but also cleaning and rinsing detergent that can create a protective and lubricative film on the surface. Ethanol, which can be used sometimes, must be avoided as it tampers the metal marking performance in a positive way, due to the formation of a temporary nanometric layer of adsorbed ethanol on the surface [22], which modifies the friction coefficient.

### 4.3. Marking Utensils

The main source of discrepancy that affects the tests repeatability and reproducibility is related to marking utensils. Each procedure uses different metallic utensils to perform metal marking test: knives are mostly used, but also forks, spoons, styli, or even spheres. In what concerns cutlery, material type (steel, silver-plated, aluminum) and composition, geometry and wear properties must be considered for the right selection. Even if they are sometimes used, the actual trend is to drop out tests with forks and spoons, as well as other materials besides steel. Above all this, the main drawback is that cutlery is not produced as standard product, meaning that no cutlery producer can supply batches of utensils that can be considered identical, considering that for example, knives production involves nearly 40 unitary operations. It is important also to consider that the knife production can suffer modifications along the time or its production could be also discontinued.

In any case, it is recommended that the selection of cutlery meet the requirements of the reference standard on stainless steel cutlery for articles in contact with food [23], in particular for a minimum hardness of 48 HRC (Rockwell Hardness scale test) requirement for the blade. It is also recommended the use of utensils that are representative of actual knife’s composition on the market. In this way, testing with both harder steel (AISI 420) and softer steel (AISI 304 or 430) is likely to provide a good information on metal marking ability in function of material hardness.

**Figure 10:**
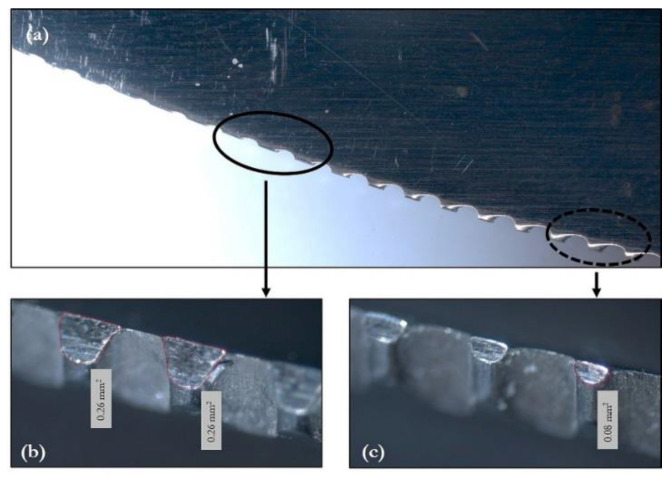
Knife wear (**a**) observed with naked eye; (**b**) heavy wear and (**c**) initial wear observed with optic microscope.

Once the knife utensil is selected, the next parameter to control is the pressure applied on the ceramic surface during a metal marking test. This parameter depends on the geometry of the serrated edge (plain or smooth edges are not normally used for these tests), the edge relative position in relation to the glazed surface, and the edge wear. The glaze’s irregularities also contribute to making it impossible to know exactly the total effective contact surface which defines the applied pressure. Figure 10 shows an AISI 420 steel graded knife with different levels of edge wear after metal marking tests. The solid line shows the region where the teeth were preferentially in contact with the glazed surface during the metal marking tests and presents relevant wear. In the dashed line, the contact area between the blade and the glaze is smaller, due to the less metal teeth wear. Moreover, it is never quite clear how many teeth are in contact with the glazed surface and therefore the effective pressure applied could not be measured. The existing protocols do not specify the degree knife’s wear qualitatively, but the use of new knives after each test should be privileged, possibly with some initial induced wear, the generated cost is a clear limitation for this practice.

In conclusion, alternative marking items based on standards materials, such as indenters, styli, or steel balls [6,7], allow a better control of applied pressure, as the contact surface is known and controllable. This practice introduces much more confidence, as reproducibility is assured.

### 4.4. Marking Operation Parameters

Even if some tests are based on manual marking realized by an operator, this practice must be dropped out for evident non-reproducibility reasons, at a moment where equipment for automatic or semi-automatic are available on the market. Metal marking testing machines are composed of an arm where the marking utensil is fixed and a support to fix the sample to be tested, in order to avoid any relative movement from the maker during the test. Depending on the model, the moving part can be either the arm or the support. The linear movements (back and forth) on the glazed surface are realized automatically or manually, and the number of back and forth movement varies from 1 to more than 10 in some cases. The linear movement can be done from rim to rim or with a specified distance. It is suggested to avoid marks to cross each other in order to prevent an excess stress and wear at crossing points that may influence a further degradation around the area and conduct to bad interpretation.

Loads placed on the arm and correctly balanced control the applied force. In a real situation, the force required to cut food ranges between 20 and 50 N [4]. In practice, in metal marking tests, the applied force ranges between 10 and 50 N, but most of them are regulated at 10 N. In terms of applied pressure, high values may be reached if just one small tooth is in contact. Several different knives were measured and it was found that the smallest tooth had 0.06 mm^2^ area, meaning that local applied pressure can reach up to more than 800 MPa, in line with values referred by Blanc [4]. It was also found that worn teeth surfaces vary from 0.06 mm^2^ to 0.50 mm^2^, and as one may consider that one to three teeth can be in contact with the surface at the same time, it means that pressure can be as local as few MPa. The combination of several scenarios (applied force between 20 and 50 N, 1 to 3 teeth in contact with the surface) with the 0.27 mm^2^ average worn area/tooth showed that an average 80 MPa is applied on the glaze.

Before evaluation, samples should be cleaned with water and detergent to remove metal particles that have not embedded in the substrate, and then dried.

### 4.5. Evaluation and Classification

One last relevant limitation of metal marking test is related to the evaluation of marks and scratches, and the consequent classification. Normally, a high level of subjectivity is associated when a visual assessment is made. Development of automatic quantification of marks through image analysis would certainly bring higher consistency to evaluation procedure. Evaluation scales are normally focused on the extreme scenarios: “no marks” and “severe marks” and on intermediate classification, such as slight or distinct marks, or even marks removed by washing. These intermediate judgments are the most difficult to assess, even with reference standards, because mark intensity [3] and its length influence the judgment. Usually two operators perform the evaluation under standardized lighting conditions, for example, according to the procedure described on washing machine resistance tests [24].

### 4.6. Metal Marking Test after Tableware Accelerated Aging

The evaluation of metal marking resistance over time, by simulation of controlled chemical degradation (washings with detergents) followed by metal marking test, is an optional procedure that permits obtaining valuable information on the durability of products, as it shows the tendency to tertiary metal making. For this, it is suggested to submit samples to an accelerating aging, according to procedure standards for domestic and hotel tableware, respectively [19,25].

## 5. CTCV Metal Marking Test Development

Based on the experience acquired on metal marking tests, CTCV has developed a new procedure, based on standardized stainless steel balls, in order to offer a test that can be widely used and to respond to the growing necessity of a trustful and uniform standard. The choice of these marking utensils was driven by the need to reduce inaccuracies and provide a better control and reproducibility. National and international producers and retailers have used the procedure since 2020.

**Figure 11:**
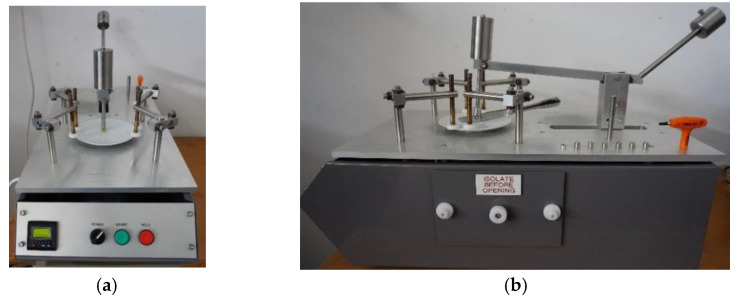
Metal marking machine (**a**) front view with steel ball test; (**b**) side view with a knife fixed to the moving arm. Manufacturer: Anderen Limited, Stoke-on-trent, UK.

**Figure 12:**
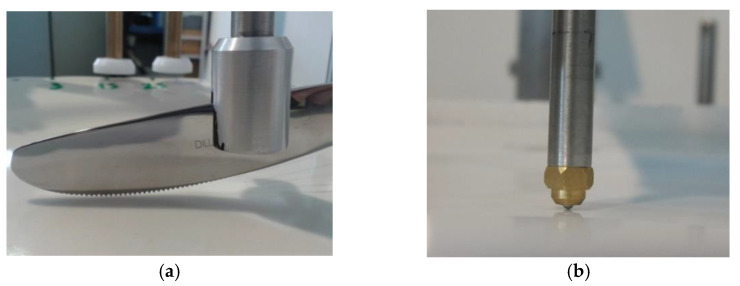
Detail on metal marking test (**a**) knife and (**b**) ø 2.5 mm stainless steel ball fixed on adapter.

With the commercial equipment (Figure 11) used at CTCV to perform the test, the ceramic tableware specimen is fixed on a stationary platform. It is possible to carry out tests using cutlery (usually knives) fixed on the moving arm, and for our purpose, a special adapter was developed with the equipment supplier to fix steel balls, as showed in showed in Figure 12. Then the length of the track, the load, and the number of back and forth movements are adjusted.

**Figure 13:**
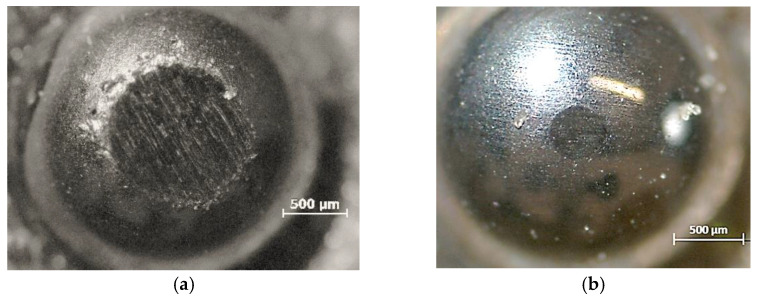
Spheres wear after the metal marking test (**a**) AISI 304 and (**b**) AISI 420.

The knife action is thus simulated by the movement of two different ø 2.5 mm standardized stainless steel balls, normally produced for mechanical application such as bearings, and thus easy to obtain on the market. AISI 420 ball with hardness 53–60 HRC and AISI 304 with hardness < 39 HRC were selected to represent most common hard and soft cutlery steel, respectively. The test is based on the principle of one ball, one specimen. The ball is fixed on the conic adapter (any rotation is avoided) and can be easily removed between each specimen. After the test, it is also possible, but not mandatory, to determine the amount of steel loosed by the ball, in order to compare the metal marking performance and the amount of steel removed from the sphere. Nevertheless, some caution must be taken because the entire amount of steel removed could not be deposited on the surface, as a part could be eliminated during the washing step, or simply not adhere to the surface. The wear of AISI 304 and AISI 420 steel spheres after a metal marking test performed on a catering dishware can be observed in Figure 13, showing an expected greater wear on the softer steel ball.

**Figure 14:**
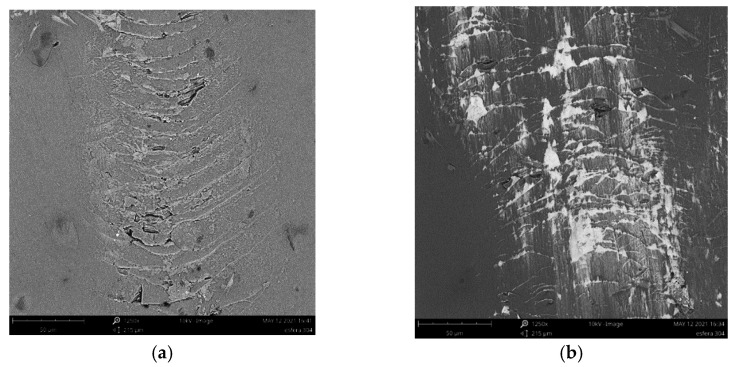
Cracks and metal deposition on glazed surface with (**a**) hard AISI420 steel and (**b**) soft AISI304 steel balls.

Scanning electron microcopy shows that the mechanical phenomenon occurring with knives is perfectly reproduced with steel balls, as shown in Figure 14.

**Figure 15:**
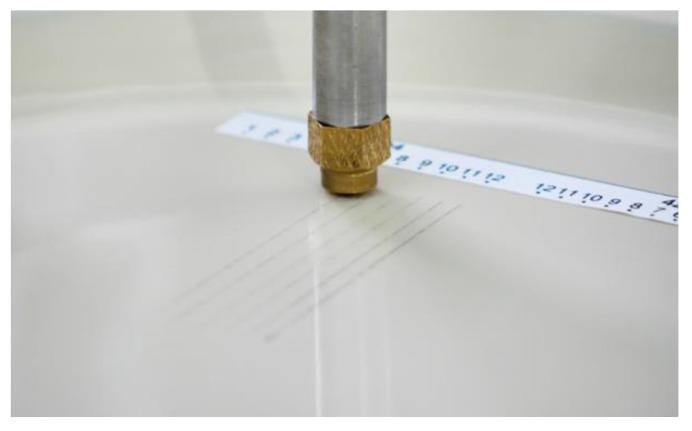
CTCV metal marking test on a porcelain dish.

The test is carried out on a sample composed of 10 specimens, with the two types of balls used on each one of them. Under a 1 kgf load, 12 parallel tracks are made (Figure 15), with 12 back and forth movements for the first one up to just one back and forth movement for the last one.

Specimens are then carefully cleaned with water and detergent, and dried. The evaluation is performed by two skilled operators, under standardized lighting conditions. An evaluation value is assigned to each individual track in function of the following mark severity criteria: 0: “no marks”, 1: “slight marks” and 2: “severe marks”.

The final classification, for both steel grades, can then be chosen following two different approaches:

- The minimum number of back and forth cycles (from 1 to 12) after which the average evaluation value of 1 is reached.
- As it was noticed that the previous classification does not always reflects the real performance of the product, a metal marking coefficient that integrates all individual evaluation value of the batch (e.g., 120 individual values per steel grade for 10 specimen) was then introduced. The coefficient C_mm_ is calculated based on a reverse cycle number weighted average of evaluation values. This parameter ranges between 0 (no marks) and 2 (severe marks in all tracks).

**Figure 16:**
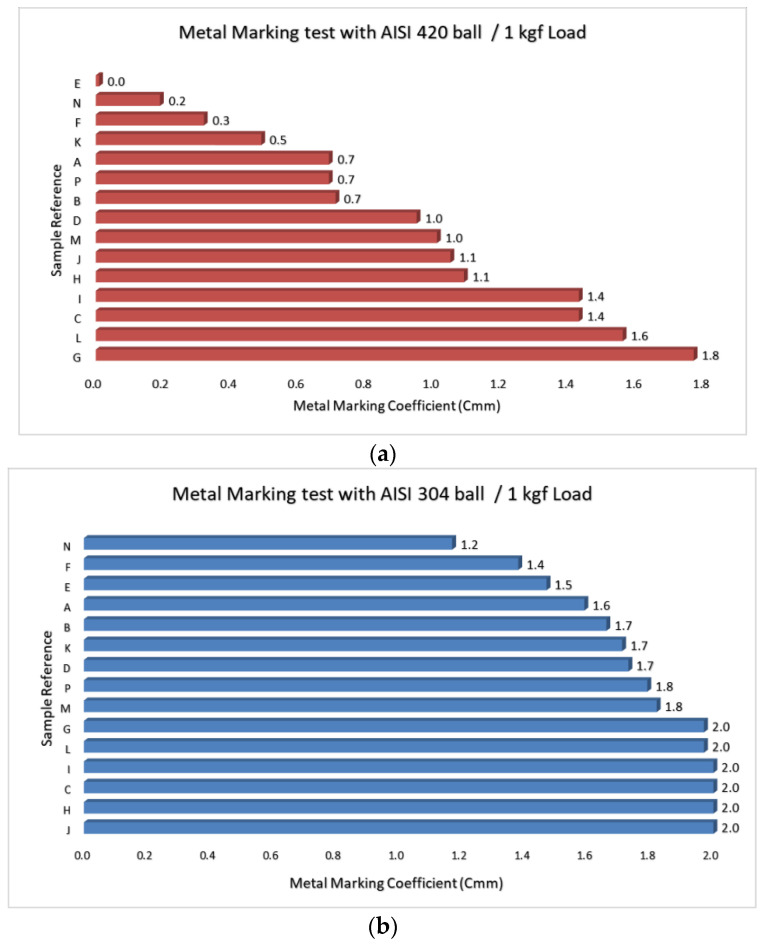
Comparative dispersion of metal marking coefficient (C_mm_) on a representative batch of Portuguese tableware products (**a**) with AISI 420 ball and (**b**) with AISI 304 ball.

Some companies are already using this procedure to control their product, allowing them to build their own database, as a wide range of in-house references are tested and compared. At the same time, studies are being made in order to optimize the testing conditions. A comparative study made on a batch of 15 different tableware products (porcelain, stoneware, and earthenware) produced by several companies. (Table 4) and Figure 16.

The objective was to test a representative range of produced tableware at national level.

The CTCV test procedure was followed, and each sample constituted of five specimens.

The analysis of results of Figure 16 shows that in the case of AISI 420 (knives steel), the metal marking coefficient cover the whole range of metal marking coefficient (from 0 to 1.8), whereas it just covers a narrow range (1.2–2.0) with AISI 304 balls (usual spoons and forks steel), with one third of tested references reaching the maximum value. We can conclude that with a softer grade, the force applied may not be adequate, especially if we consider that in real conditions of use, higher forces are applied with knives than with forks or spoons. Thus, we propose to study the application of a lower force on the tableware with this softer grade, in order to be also in line with real use of utensil. Collected data from private testing realized with our customers corroborate that a wider range of C_mm_ is achieved when testing with 0.4 kgf compared to 1 kgf. The objective is now to corroborate these results with metal marking performance of all these products in their normal daily use. To achieve this, we maintain a straight collaboration with companies, in order to analyze eventual reclamation from end-users.

## 6. Conclusions

Metal marking is a long-time transversal problem of ceramic tableware industry sector that deeply affects the product performance. The research and development work done by raw materials and ceramic producers to develop glazes with better performance in terms of metal marking is being embraced: extremely low roughness glazes, reduction of crystalline phases, chemical resistance, harder glazes, application of protective films or even replication of glazes with excellent metal marking performance. Despite all these efforts and also the use and care recommendation provided to users, it is impossible to control the conditions of service, the type of materials and the quality of the cutlery, the chemical detergents aggressiveness, the washing conditions, and the severe handling conditions typical of the hospitality industry.

The actual lack of recognized standards, explained by the recurrent difficulties to establish reliable test protocols, represents an obvious limitation for the comparison between products, as existing tests do not guarantee trustworthy and reproducible results. It is expected that the recent test developed at CTCV can establish an alternative foreseeing the harmonization of metal marking performance tests. Future development works will aim to establish the effective relationship between laboratory test results and metal marking occurrence in real conditions of use.

## Figures and Tables

**Table 1:** Glazes parameters influencing metal marking behavior.

Glaze Surface Properties	Glaze Mechanical Properties	Glaze Composition
Profile	Young modulus	Ratio vitreous matrix/crystalline phases
Roughness	Hardness	Hard crystalline phases composition
Microdefects presence (cracks, bubbles, impurities)	Thermal expansion coefficient (glaze and body)	Recrystallization during firing and cooling

**Table 2:** Cutlery (especially knives) parameters influencing metal marking behavior.

Geometric Properties	Mechanical Properties	Composition	Production
Type of blade and edge	Hardness	Alloy	Quality control
Teeth sharpness	Wear behavior	Type of steel	Heat treatment

**Table 3:** General methods to improve metal marking resistance.

Technological Solution/Action	Advantages and Limitations
Replication of performant glaze recipes	Limited knowledge of underlying reasons for good behavior.
Firing cycle optimization	Reduction of crystalline particles in the glaze, but local temperature variations in the furnace may limit the effect on some parts, especially in older furnaces.
Glaze composition with reduction/modification of crystalline phase (particle size, alternative opacifying materials, etc.).	Requires long and expensive testing, generally limited by tight physical and aspect variation range.
Increase of compression stress of the glaze	Higher stress is needed to provoke cracking. However, peeling must be avoided.
Reduction of glaze roughness	Wide range of possible action from lower melting components and reduction of particle size of crystals.
Protection of glaze with coatings	Challenging materials research and development. Good potential of dissemination of developed solution. Final aspect and cost increase barrier Possible coating/glaze mismatch.

**Table 4:** List of tested tableware bodies and glazes.

Sample	Body	Glaze Characteristics		
		Opacity	Surface Finish	Colour
A	Porcelain	Transparent	Gloss	White
B	Porcelain	Transparent	Gloss	White
C	Fine Stoneware	Opaque	Matte	White
D	Fine Stoneware	Opaque	Gloss	Blue
E	Fine Stoneware	Opaque	Matte	Grey
F	Fine Stoneware	Opaque	Gloss	Green
G	Fine Stoneware	Opaque	Gloss	White
H	Fine Stoneware	Opaque	Matte	Beige
I	Earthenware	Opaque	Matte	Cream
J	Earthenware	Opaque	Gloss	White
K	Porcelain	Transparent	Gloss	White
L	Earthenware	Opaque	Gloss	White
M	Earthenware	Transparent	Gloss	Cream
N	Earthenware	Opaque	Gloss	Rose
P	Earthenware	Transparent	Gloss	Cream

## References

[1] Castilone R., Carty W. (1997). The metal marking behavior of matte, gloss and zircon-opacified glazes. Ceram. Eng. Sci. Proc..

[2] Lee H., Carty W., Castilone R. (2004). Metal marking of dinnerware glaze: Correlation with friction and surface roughness. Ceram. Eng. Sci. Proc..

[3] Seedorff Z., Patterson R., Pangels H. (1992). Testing metal marking resistance. Ceram. Eng. Sci. Proc..

[4] Blanc J. (2003). Méthode d’essais de marquage des assiettes par les couteaux. L’Ind. Céram. Ver..

[5] Güngör F., Altun B. (2018). Development of scratch resistance of soft porcelain opaque glazes. Trans. Ind. Ceram. Soc..

[6] Pee J., Lee N., Kim G., Kim Y., Oh Y., Kim H., Kim G. (2016). Effect of frit content on the metal marking and scratching resistance of celadon glaze. Key Eng. Mat..

[7] (2015). Domestic and Hospitality Use Ceramic Tableware Articles Intended for Contact with Foodstuffs.

[8] Geller R., Creamer A. (1931). Metal marking of whiteware glazes as influenced by sulphur and carbon in kiln atmospheres. J. Am. Ceram. Soc..

[9] Castilone R., Sriram D., Carty W. (1999). Crystallization of zircon in stoneware glazes. J. Am. Ceram. Soc..

[10] Prieur C., Bisson G., Casset C. (1997). Marquage de la vaisselle céramique par les couverts. L’Ind. Céram. Ver..

[11] Plesingerova B., Kovalcikova M. (2003). Influence of the thermal expansion mismatch between body and glaze on the crack density of glazed ceramics. Ceram.-Silik..

[12] Cannillo V., Esposito L., Rambaldi E., Sola A., Tucci A. (2009). Microstructural and mechanical changes by chemical ageing of glazed ceramic surfaces. J. Eur. Ceram. Soc..

[13] Heo S., Kim S., Kim U., Pee J., Han Y., Kim S., Lee S., Kim H., Oh Y. (2015). Tribological behavior of whiteware with different transparent glazes. J. Korean Ceram. Soc..

[14] Wang S., Peng C., Xiao H., Wu J. (2015). Microstructural evolution and crystallization mechanism of zircon from frit glaze. J. Eur. Ceram. Soc..

[15] Wang S., Peng C., Huang Z., Zhou J., Lu M., Wu J. (2014). Clustering of zircon in raw glaze and its influence on optical properties of opaque glaze. J. Eur. Ceram. Soc..

[16] Garrido L.B., Aglietti E.F., Martorello L., Camerucci M.A., Cavalieri A.L. (2006). Hardness and fracture toughness of mullite-zirconia composite obtained by slip casting. Mater. Sci. Eng. A.

[17] Whitney D.L., Broz M., Cook R.F. (2007). Hardness, toughness, and modulus of some common metamorphic minerals. Am. Mineral..

[18] Karlsson S., Iwasa M. (2000). Evaluation of chemical corrosion of tableware glazes by atomic force microscopy. Ceram. Forum Int..

[19] (2006). Mechanical Dishwashing Resistance of Utensils—Part 4: Rapid Test for Domestic Ceramic Articles.

[20] Sheikhattar M., Attar H., Sharafi S., Carty W. (2016). Influence of surface crystallinity on the surface roughness of different ceramic glazes. Mater. Charact..

[21] Karlsson S., Linde K., Carsson R. (1991). On the influence of glaze properties on glaze damage. Euro-Ceram. II.

[22] Kurihara K., Mizukami M. (2007). Hydrogen bonded molecular macrocluster formation on silica surfaces in non-polar solvents. Rep. Inst. Fluid Sci..

[23] (1997). Materials and Articles in Contact with Foodstuffs—Cutlery and Table Hollowware. Part 2: Requirements for Stainless Steel and Silver-Plated Cutlery.

[24] (2001). Mechanical Dishwashing Resistance of Utensils—Part 2: Inspection of Non-Metallic Articles.

[25] (2006). Mechanical Dishwashing Resistance of Utensils—Part 5: Rapid Test for Catering Ceramic Articles.

